# Signatures of natural selection between life cycle stages separated by metamorphosis in European eel

**DOI:** 10.1186/s12864-015-1754-3

**Published:** 2015-08-13

**Authors:** J. M. Pujolar, M. W. Jacobsen, D. Bekkevold, J. Lobón-Cervià, B. Jónsson, L. Bernatchez, M. M. Hansen

**Affiliations:** Department of Bioscience, Aarhus University, Aarhus C, Aarhus, Denmark; National Institute of Aquatic Resources, Technical University of Denmark, Silkeborg, Denmark; National Museum of Natural Sciences (CSIC), Madrid, Spain; Biopol, Marine Biology and Biotechnology Center, Skagastrond, Iceland; IBIS (Institut de Biologie Intégrative et des Systèmes), Université Laval, Québec, Canada

**Keywords:** Adaptative decoupling hypothesis, Complex life cycles, Metamorphosis, RAD sequencing, Selection

## Abstract

**Background:**

Species showing complex life cycles provide excellent opportunities to study the genetic associations between life cycle stages, as selective pressures may differ before and after metamorphosis. The European eel presents a complex life cycle with two metamorphoses, a first metamorphosis from larvae into glass eels (juvenile stage) and a second metamorphosis into silver eels (adult stage). We tested the hypothesis that different genes and gene pathways will be under selection at different life stages when comparing the genetic associations between glass eels and silver eels.

**Results:**

We used two sets of markers to test for selection: first, we genotyped individuals using a panel of 80 coding-gene single nucleotide polymorphisms (SNPs) developed in American eel; second, we investigated selection at the genome level using a total of 153,423 RAD-sequencing generated SNPs widely distributed across the genome. Using the RAD approach, outlier tests identified a total of 2413 (1.57 %) potentially selected SNPs. Functional annotation analysis identified signal transduction pathways as the most over-represented group of genes, including MAPK/Erk signalling, calcium signalling and GnRH (gonadotropin-releasing hormone) signalling. Many of the over-represented pathways were related to growth, while others could result from the different conditions that eels inhabit during their life cycle.

**Conclusions:**

The observation of different genes and gene pathways under selection when comparing glass eels vs. silver eels supports the adaptive decoupling hypothesis for the benefits of metamorphosis. Partitioning the life cycle into discrete morphological phases may be overall beneficial since it allows the different life stages to respond independently to their unique selection pressures. This might translate into a more effective use of food and niche resources and/or performance of phase-specific tasks (e.g. feeding in the case of glass eels, migrating and reproducing in the case of silver eels).

**Electronic supplementary material:**

The online version of this article (doi:10.1186/s12864-015-1754-3) contains supplementary material, which is available to authorized users.

## Background

Many animals show complex life cycles organized into morphologically distinct phases separated by abrupt metamorphic transitions (metamorphosis), as opposed to single static or continuously changing phases [1]. Complex life cycles are ubiquitous in nature and have evolved many times independently [1–3]. Life stages are believed to represent alternative adaptations for optimal food and niche exploitation as well as conflicting tasks (e.g. feeding, growth, mate-finding, dispersal, reproduction). Since Darwin, evolutionary biologists have been interested in understanding the genetic associations between life cycle stages and to what extent discrete phases are free to evolve independently from one another. Metamorphosis marks drastic morphological, physiological, behavioural and ecological changes in the life cycles of animals [4]. Given the dramatic changes associated with metamorphosis, selection could differ before and after metamorphosis, and opposing selection might be more common than complimentary selection [5, 6]. In regard to the benefits of metamorphosis, the adaptive decoupling hypothesis [1] predicts that traits separated by metamorphosis should be genetically uncorrelated, allowing distinct phases to respond independently to different selective forces, without correlated negative effects on traits of alternative phases. Studies testing the adaptive decoupling hypothesis have found contradictory results and the genetic associations between life cycle stages separated by metamorphosis remain poorly understood [6].

Our species of interest is the European eel (*Anguilla anguilla*), a facultative catadromous fish with a particularly complex life cycle that includes two metamorphoses. After spawning in the remote Sargasso Sea, larvae of European eel are transported to the coasts of Europe and North Africa following the Gulf Stream and North Atlantic current. On reaching the continental shelf, eels undergo a first metamorphosis from larvae into glass eels (juvenile stage), which complete their migration into continental feeding habitats as yellow eels. After an extensive feeding/growing period, eels undergo a second metamorphosis into silver eels (adult stage). The so-called “silvering metamorphosis” encompasses morphological (colour, eye size, body length and weight) as well as physiological modifications (e.g. loss of digestive tract), together with the development of gonads. These changes are beforehand preparation for the future spawning migration back to the Sargasso Sea, where eels reproduce and die [7].

The European eel is an ideal species in which to study local selection. First, it presents a large effective population size (estimated from 100,000 to 1×10^6^ individuals; [8]) that renders natural selection the major force determining genetic differences; hence the role of random genetic drift is expected to be negligible. Second, it is present across extremely heterogenous environments in terms of temperature (from subarctic habitats in Iceland, Norway and northwestern Russia to subtropical habitats in North Africa and the Mediterranean Sea), salinity (from fresh water to brackish and marine habitats), substrate, depth and productivity along its geographic distribution [9]. Despite such a wide distribution range, there is recent conclusive evidence for panmixia in European eel, i.e. the existence of a single randomly mating population. In the most comprehensive study to date genotyping over 1000 individuals obtained throughout all the distribution range in Europe at 21 microsatellite loci, Als et al. [10] showed a very low and nonsignificant genetic diffferentiation across Europe (F_ST_ = 0.00024) and a lack of substructuring among larvae collected in the Sargasso Sea (F_ST_ = 0.00076). Moreover, no significant genetic differentiation was observed when comparing the samples obtained in Europe vs. the larvae obtained from the spawning area (F_ST_ = -0.00012). Panmixia was also confirmed at the genomic level in a study using a large dataset of > 450,000 SNPs from 259 RAD-sequenced European eels [11], which showed low levels of genetic differentiation (F_ST_ = 0.0007). Previous studies based on cohort analysis showed an unpatterned genetic heterogeneity (genetic patchiness) as samples did not group together according to sampling location or cohort, and consequently no pattern on Isolation-by-Distance or Isolation-by-Time was detected [12, 13]. If European eel larvae showed phylopatry to the parental original freshwater habitats, genetic differences would be expected across Europe; hence, the lack of genetic structuring found suggest no larval homing and random larval migratory routes [10, 11]. One consequence of panmixia and random dispersal of larvae across habitats is that long-term local adaptation is not possible in eels, despite the high potential for selective responses due to high mortalities in both early and late life stages [14]. Any signature of spatially varying selection in a given generation is expected to be lost in the subsequent generation, preventing heritable trans-generational local adaptation [15]. However, single-generation signatures of local selection are still detectable [11, 15].

Studies of adaptive evolution in European eel have focused on the detection of signatures of local selection in glass eels. Using a panel of 100 coding-gene single nucleotide polymorphisms (SNPs), Ulrik et al. [16] found signatures of selection at 11 loci in European eel, which constituted genes with a role in metabolism as well as defense response. As an alternative to candidate gene approaches, Pujolar et al. [11] tested for footprints of selection in glass eels at the genome level using 50,354 SNPs generated by RAD sequencing. A total of 754 potentially locally selected SNPs were identified. Candidate genes for local selection constituted a wide array of functions, including calcium signalling, neuroactive ligand-receptor interaction and circadian rhythm.

The power and efficiency of next generation sequencing (NGS) technologies are enabling research use of genomic data to address ecological and evolutionary questions at a genome-wide scale for model and non-model species [17, 18]. The insights that are obtained through NGS methods, as well as high throughput genotyping methods in general, have led to unprecedented progress in many areas, from bridging ecological and evolutionary concepts to identifying the molecular basis of local selection and adaptation [17, 19–24]. The aim of our study is to test for selection acting upon different life cycle stages separated by metamorphosis in European eel, in particular between glass eels (juvenile stage) and silver eels (adult stage). First, we used a candidate gene approach and genotyped individuals from three sampling locations (Iceland, Ireland and Spain) using a panel of 100 coding-gene SNPs. Second, we performed RAD-sequencing of individuals from two sampling locations (Ireland and Spain), which allowed us to test for signatures of local selection at the genome level using a total of 153,423 SNPs widely distributed across the genome. Following the shifts in selection exerted by the dramatic physiological, morphological and ecological changes that accompany metamorphosis, our prediction is that different genes and gene pathways will be under selection at different life stages, and those will appear as outlier loci (higher genetic differentiation relative to the background) when comparing glass eels vs. silver eels. Ultimately, the identification of genes showing marked differences when comparing life stages separated by metamorphosis can provide insights into how European eel can cope with the incredible variety of conditions and environments encountered throughout its complex life cycle. It can also provide general insights into adaptive genomic evolution in natural populations of marine fishes.

## Results

### SNP genotyping

Details of all polymorphic loci, including frequency of the most common allele across all sampling locations in our study in silver eels and glass eels are presented in Table 1. A total of 61 out of 80 SNPs were polymorphic, although at 19 loci minor allele frequency (MAF) was < 0.05 in all samples. Genetic diversity values are summarized in Table 2. Genetic diversity measures were very similar across samples and no significant differences were found across sampling locations in silver eels, i.e. H_e_ (Spain: 0.227; Ireland: 0.233; Iceland: 0.224; p = 0.96); AR (Spain: 1.60; Ireland: 1.62; Iceland: 1.62; p = 0.99). Moreover, no differences were found in genetic diversity measures when silver eels were compared to glass eels (p > 0.05). Three loci deviated significantly from HWE at all locations (UGP2, heterozygote deficit; TENT7and UGPA, heterozygote excess) but were not excluded from the analysis, since departures from HWE might be due to selection.

**Table 1:** Details of all polymorphic loci, including frequency of the most common allele across all silver eel (SE) and glass eel (GE) samples

Locus	Gene	Ireland		Iceland		Spain	
		SE	GE	SE	GE	SE	GE
40S_S18_1401	40s ribosomal protein s18	0.829	0.846	0.828	0.807	0.838	0.750
60S_L10A_21874	60s ribosomal protein L10a	0.846	0.782	0.776	0.839	0.775	0.779
ACT_A3B_8646	Actinin alpha 3b	1.000	1.000	1.000	1.000	1.000	1.000
ACTB_21752	Beta-actin	0.788	0.808	0.906	0.862	0.863	0.886
ACYL_13914	Acyl carrier protein	0.750	0.776	0.758	0.823	0.738	0.826
ADH_3	Alcohol dehydrogenase class-3	0.737	0.859	0.733	0.690	0.795	0.761
ADSS_L1_15447	Adenylosuccinate synthetase isozyme 1	1.000	0.987	1.000	0.984	1.000	1.000
ALD_R	Aldose reductase	0.759	0.838	---	0.537	0.859	0.936
ALDH_2_16634	Aldehyde dehydrogenase 2	0.638	0.763	0.600	0.550	0.688	0.682
ANK_R_13478	Ankyrin repeat domain-comtaining protein 1	1.000	1.000	1.000	1.000	1.000	1.000
ANN_A11_16176	Annexin A11	0.897	0.923	0.807	0.919	0.900	0.852
ANX_2_249	Annexin A2-A	0.987	0.949	1.000	0.983	0.975	1.000
ARF_4_18099	ADP-ribsylation factor 4	0.987	1.000	0.969	0.983	0.988	0.977
ATP_BC_259	ATP-bindincasette sub-family A member 1	1.000	1.000	1.000	1.000	0.988	0.989
BPNT_1_18778	3'(5'),5'-biphosphate nucleotidase 1	0.971	1.000	0.985	1.000	1.000	1.000
CLIC_5_10148	Chloride intracellular channel 5	0.568	0.487	0.521	0.492	0.521	0.492
COI_17591	Cytochrome oxidase subunit I	1.000	1.000	1.000	1.000	1.000	1.000
COP_9_18132	266S protease regulatory subunit 7	0.950	0.962	0.968	0.968	0.988	0.966
CSDE_1_11069	Cold shock domain-containing protein E1	1.000	1.000	1.000	1.000	1.000	1.000
CSDE_1_19713	Cold shock domain-containing protein E1	0.950	1.000	0.968	0.983	0.963	0.977
CST_21113	Cystatin precursor	0.782	0.597	0.726	0.661	0.775	0.750
CYT_BC1_9061	Cytochrome b-c1 complex subunit 2	1.000	1.000	1.000	1.000	1.000	1.000
EF_1G_4796	Translation elongation factor 1 gamma	1.000	1.000	1.000	1.000	1.000	1.000
EF2_10494	Translation elongation factor 2	1.000	1.000	1.000	1.000	1.000	1.000
EIF_3F_341	Translation elongation factor 3 subunit F	0.872	0.892	0.967	0.967	0.910	0.932
EIF_3J_11587	Translation elongation factor 3 subunit J	0.963	0.974	0.940	0.968	0.950	0.930
FER_H_20955	Ferritin heavy subunit	0.988	1.000	1.000	0.983	1.000	1.000
FGB_47	Fibrinogen Beta Chain	1.000	1.000	1.000	1.000	1.000	1.000
GAPDH_20355	Glyceraldehyde-3-phoshpate dehydrogenase	0.962	0.962	0.969	0.589	0.974	1.000
GDE1_2508	Glycerophosphochlorine phosphodiesterase	1.000	1.000	1.000	1.000	1.000	1.000
GOG_B1_15792	Golgin sub-family B member 1	0.987	0.987	0.970	0.968	1.000	0.966
GPX_4_19607	Glutathione peroxidase 4	1.000	1.000	1.000	1.000	1.000	1.000
HMG_T_9973	High mobility group-T protein	0.988	0.988	1.000	0.984	0.988	0.966
HSP_90A_15666	Heat shock protein 90 alpha	0.988	0.949	0.984	0.968	0.988	0.977
HSP_90B_21100	Heat shock protein 90 beta	0.895	0.949	0.966	0.984	1.000	1.000
HSPE_1_17854	10 kDa heat shock protein	1.000	1.000	1.000	0.984	1.000	1.000
IF_RF2_19747	Interferon regulatory factor 2	1.000	1.000	1.000	1.000	1.000	1.000
JAM_3_13916	Junctional adhesion molecule 3b	0.938	0.885	0.984	0.952	0.950	0.893
KRT_13_20618	Keratin	0.808	0.795	0.821	0.783	0.800	0.796
KRT_A_15738	Keratin alpha-like	0.975	1.000	0.952	0.984	0.960	0.988
LBL_L2_20921	No hit	1.000	1.000	1.000	1.000	1.000	1.000
LDH_B_9441	Lactase dehydrogenase B	1.000	1.000	0.952	0.950	1.000	1.000
MDH_1393	Malate dehydrogenase	0.449	0.592	0.567	0.603	0.615	0.476
MYH_14857	Superfast myosin heavy chain	0.514	0.473	0.500	0.516	0.425	0.489
NADH_4_21742	NADH dehydrogenase subunit 4	1.000	1.000	1.000	1.000	1.000	1.000
NADH_5_17101	NADH dehydrogenase subunit 5	0.974	1.000	1.000	1.000	1.000	0.977
NADH1_10_21119	NADH dehydrogenase 1 alpha subunit 10	1.000	1.000	1.000	1.000	1.000	1.000
NCP_2_15547	Nucleolar complex protein 2	0.974	0.934	1.000	0.967	0.962	0.932
NEX_19953	Nexilin	0.592	0.608	0.485	0.567	0.577	0.523
NGD_21138	Neuroguidin	0.795	0.842	0.906	0.758	0.825	0.852
NRAP_1541	Nebulin-related anchoring protein	1.000	0.987	0.984	1.000	0.988	0.989
PA2G4_2600	Proliferation associated protein 2G4	0.663	0.645	0.652	0.550	0.575	0.580
PFN_15113	Profilin-2	1.000	1.000	1.000	1.000	1.000	1.000
PGD_18096	6-phosphogluconate dehydrogenase	0.987	0.961	0.969	0.967	0.988	0.966
PGI_1	Phosphoglucose isomerase-1	0.800	0.797	0.742	0.750	0.675	0.807
PGI_2	Phosphoglucose isomerase-2	0.552	0.566	0.553	0.500	0.625	0.556
PGK_1_11454	Phosphoglycerate kinase 1	0.947	0.961	0.909	0.903	0.975	0.939
PRP_40_16504	Pre-mRNA-processing factor 40 homolog A	0.829	0.842	0.833	0.903	0.846	0.845
PSA_4_21534	Proteasome subunit alpha type-4	0.513	0.434	0.550	0.550	0.590	0.432
PSME_1_21196	Proteasome activator	0.719	0.583	-	0.621	0.625	0.667
RFC_3_18186	Replication factor C subunit 3	0.763	0.776	0.773	0.717	0.800	0.784
RTF_1_21288	RNA polymerase-associated protein RTF1	0.756	0.724	0.900	0.758	0.846	0.750
SDH_O	Sorbitol dehydrogenase	1.000	1.000	1.000	1.000	1.000	1.000
SLC_25A5_19808	ADP/ATP translocase 2	1.000	1.000	0.967	0.984	1.000	1.000
SM_22_6449	Transgelin	0.963	0.934	0.946	0.952	0.950	0.955
SN4_TDR_374	Taphylococcal nuclease domain-containing protein	0.962	0.910	0.919	0.862	0.888	0.909
TENT_02_11046	No hit	0.744	0.711	0.739	0.774	0.713	0.784
TENT_03_12589	Collagen type XXVIII alpha 1 a	0.949	0.987	1.000	0.968	0.988	0.966
TENT_05_19704	No hit	1.000	0.987	0.968	0.983	1.000	1.000
TENT_06_16512	Protein Phosphatase regulatory subunit	0.603	0.581	0.600	0.533	0.575	0.625
TENT_07_21161	No hit	0.419	0.368	0.348	0.397	0.350	0.286
TNNT_2E_20968	Troponin T2e	0.949	0.885	0.913	0.875	0.838	0.895
TRIM_35_8416	Tripartite motif-contaning protein 35	0.692	0.658	0.750	0.629	0.641	0.698
TTN_B_20952	Titin b	1.000	1.000	1.000	1.000	1.000	1.000
TUB_A_19211	Tubulin alpha 2	1.000	1.000	1.000	1.000	1.000	1.000
UBI_A52_5049	Ubiquitin A-52 ribosomal protein fusion product 1	0.987	0.974	0.967	0.917	1.000	0.989
UGP_2_2128	UDP-glucose pyrophosphorylase 2	0.671	0.541	0.531	0.690	0.663	0.546
UGP_A_2307	Glycerol-3-phosphate transporter subunit	0.671	0.603	0.600	0.613	0.600	0.636
UNA_SINE2_16912	Eel Short interspersed elements	1.000	1.000	1.000	1.000	1.000	1.000
ZETA_15177	Tyr 3-monooxygenase	0.739	0.865	0.810	0.783	0.756	0.852

**Table 2:** Summary of genetic diversity indices at 80 SNPs including observed (H_o_) and expected heterozygosities (H_e_), polymorphism at the 95 % and 99 % level, mean (MNA) and total number of alleles (TNA) and allelic richness (AR) in all silver eel (SE) and glass eel (GE) samples

	N	H_o_	H_e_	P_95_	P_99_	MNA	TNA	AR
Spain-SE	40	0.232	0.227	0.625	0.844	1.84	118	1.60
Spain-GE	44	0.224	0.228	0.641	0.859	1.86	119	1.63
Ireland-SE	40	0.226	0.233	0.641	0.891	1.89	121	1.62
Ireland-GE	39	0.236	0.233	0.656	0.844	1.84	118	1.62
Iceland-SE	33	0.222	0.224	0.581	0.855	1.86	119	1.62
Iceland-GE	40	0.272	0.249	0.625	0.938	1.94	124	1.66

Genetic differentiation across silver eel samples was low and not significant (F_ST_ = 0.0018; p = 0.330). A similar low genetic differentiation was found when considering all silver eel and glass eel samples (F_ST_ = 0.0026; p = 0.079) or when comparing pooled silver eels vs. pooled glass eels (F_ST_ = 0.0021; p = 0.807). Low F_ST_ values were also obtained when compared silver eels vs. glass eels in all samples: Spain (F_ST_ = 0.0036; p = 0.851), Ireland (F_ST_ = 0.0016; p = 0.943) or Iceland (F_ST_ = 0.0065; p = 0.976).

Prior to the selection analysis, we investigated the presence of hybrids in the silver eel data set using STRUCTURE. Two individuals from Iceland (VADA-1 and VADA-2) were identified as hybrids showing a 50 % admixture proportion, and were consequently removed from the analysis (data not shown).

Results from outlier tests are summarized in Table 3. Using LOSITAN, a total of 3 outliers were identified when comparing all samples in our study (3 silver eels, 3 glass eels): GAPDH, ALD_R and CLIC5. When comparing the 3 silver eel samples, two outliers were identified: CLIC5 and LDHB. When comparing silver eels vs. glass eels in all samples separately, different outlier loci were identified at each location: CLIC5 when comparing silver eels and glass eels from Spain; CST and CSDE1 when comparing silver eels and glass eels from Ireland; and GAPDH when comparing silver eels and glass eels from Iceland. GAPDH was also detected as outlier when comparing pooled silver eels vs. pooled glass eels. When using BAYESCAN, fewer outliers were identified in comparison with LOSITAN, all showing high F_ST_ values: GAPDH (F_ST_ = 0.27) and ALD_R (F_ST_ = 0.12) when considering all samples and GAPDH (F_ST_ = 0.33) when comparing silver eels and glass eels from Iceland.

**Table 3:** Candidate genes under selection at 80 SNPs in LOSITAN and BAYESCAN. SE = Silver eels; GE = Glass eels

Samples	LOSITAN	BAYESCAN
All samples (SE + GE)	ALD_R	ALD-R
	(H_e_ = 0.34; F_ST_ = 0.12; p = 0.001)	(BPP = 1.00; q = 0.000; alpha = 2.1)
	GAPDH	GAPDH
	(H_e_ = 0.17; F_ST_ = 0.27; p = 0.001)	(BPP = 1.00; q = 0.000; alpha = 2.6)
	CLIC_5_10148	
	(H_e_ = 0.50; F_ST_ = 0.05; p = 0.009)	
Silver eels (SE)	CLIC_5_10148	
	(H_e_ = 0.51; F_ST_ = 0.09; p = 0.003)	
	LDH_B_9441	
	(H_e_ = 0.03; F_ST_ = 0.03; p = 0.047)	
SE(pooled) vs. GE(pooled)	GAPDH	
	(H_e_ = 0.14; F_ST_ = 0.05; p = 0.010)	
Spain (SE vs. GE)	CLIC_5_10148	
	(H_e_ = 0.51; F_ST_ = 0.08; p = 0.018)	
Ireland (SE vs. GE)	CST_21113	
	(H_e_ = 0.44; F_ST_ = 0.07; p = 0.023)	
	CSDE_1_19713	
	(H_e_ = 0.05; F_ST_ = 0.04; p = 0.049)	
Iceland (SE vs. GE)	GAPDH	GAPDH
	(H_e_ = 0.41; F_ST_ = 0.33; p = 0.001)	(BPP = 1.00; q = 0.000; alpha = 2.4)

### RAD sequencing

After sequencing of the RAD libraries, average number of reads (90 bp) per individual was 12.2 million for silver eels and 9.6 million for glass eels. After trimming to 75 bp and quality filtered, the average number of high quality reads retained was 10.7 million (86.8 %) for silver eels and 7.9 million (82.2 %) for glass eels. A similar percentage of reads were uniquely aligned to the European eel draft genome (69.3-70.0 %), while 25.6-26.3 % of sequences did not align and 4.5 % were discarded due to multiple alignments.

Aligned reads were then assembled into a total of 348,342 loci using Stacks (Table 4). After discarding 27.12 % of loci due to insufficient coverage, 253,864 loci were used to construct a catalog of loci of SNPs for all individuals. At this point, a more strict filtering was applied and we eliminated 846 loci due to extremely high coverage (>57.3 reads, which is twice the standard deviation from the mean number of reads), 144 loci at which all individuals were either all heterozygotes or all homozygotes, and 55,075 loci due to the presence of more than 2 alleles in a single individual, possibly reflecting paralogs or sequencing error. After a final filtering step selecting only loci genotyped in >66.7 % of individuals in all sampling locations, a total of 77,337 RAD loci were retained. Using Populations in Stacks, a total of 558,022 SNPs were discovered, including 153,423 SNPs with a minor allele frequency > 0.05.

**Table 4:** Statistics describing the distribution of different properties of RAD sequences after each step of filtering (FASTX-Toolkit), alignment to the eel draft genome (BOWTIE) and assemblage into loci (STACKS) in silver eels (SE) and glass eels (GE)

FASTX											
Group	Raw reads	Filtered reads	% Eliminated	Mean Q	Q1	Med	Q3	% A	% C	% G	% T
SE	12234656	10692256	13.2	38.7	38	39.7	40	28.7	22.0	20.4	28.7
GE	9593701	7899505	17.8	38.7	38	39.4	40.2	29.5	20.7	20.4	29.4
BOWTIE											
Group	Reads	Aligned	% Aligned	Non-aligned		% Non-aligned		Discarded		% Discarded	
SE	10692256	7433827	69.3	2782716		26.3		475668		4.5	
GE	7899505	5527660	70.0	2018833		26.0		353043		4.5	
STACKS											
Group	Reads	Stacks	Loci	Loci used		% Loci used		Loci discarded		% Loci discarded	
SE	7433827	547017	348342	253864		73.9		94482		27.1	
GE	5527660	526821	335343	217326		64.5		118016		35.5	

Measures of genetic diversity at 77,337 loci at all sampling locations are summarized in Table 5. The Valencia silver eel sample showed similar heterozygosities (H_o_ = 0.048; H_e_ = 0.052) and nucleotide diversity (Pi = 0.053) compared to the Valencia glass eel sample (H_o_ = 0.047; H_e_ = 0.051; Pi = 0.052) and the Burrishoole glass eel sample (H_o_ = 0.047; H_e_ = 0.051; Pi = 0.052). The Burrishoole silver eel sample showed slightly lower diversity (H_o_ = 0.040; H_e_ = 0.042; Pi = 0.045), presumably due to lower sample size.

**Table 5:** Summary of genetic diversity indices at 77,337 RAD-loci including observed (H_o_) and expected heterozygosities (H_e_) and nucleotide diversity (Pi) considering only variant positions and considering all positions in all silver eel (SE) and glass eel (GE) samples

	N	Variant positions			All positions		
		H_o_	H_e_	Pi	H_o_	H_e_	Pi
Spain-SE	31	0.0479	0.0523	0.0532	0.0046	0.0050	0.0051
Spain-GE	31	0.0465	0.0508	0.0518	0.0044	0.0048	0.0049
Ireland-SE	10	0.0401	0.0420	0.0448	0.0039	0.0041	0.0043
Ireland-GE	29	0.0469	0.0505	0.0515	0.0045	0.0048	0.0049

Prior to the analysis of local selection, the presence of hybrids in the data set was assessed in STRUCTURE using a subset of diagnostic SNPs between North Atlantic eels and previously analyzed RAD-sequenced American eels for comparison. A scenario with K = 2 groups corresponding to the two North Atlantic eel species was suggested. Within European eel, all RAD-sequenced silver eels were identified as pure European eel, with no hybrids in the data set.

In the RAD data set, outlier tests were first conducted comparing silver eels (N = 31) and glass eels (N = 31) from Valencia using 153,423 SNPs. A total of 2413 SNPs putatively under selection were identified by LOSITAN and 1472 SNPs by BAYESCAN. Since the LOSITAN outliers encompassed those outliers identified by BAYESCAN, the rest of the analysis was conducted only for the LOSITAN outliers.

Out of the 2413 candidate SNPs, a hit with a gene was obtained for 1089 (45.13 %) of the SNPs, while the remaining 1324 SNPs (54.87 %) were located in noncoding regions of the genome. Among hits, 966 were located in introns and 123 in exons, including 48 in complete coding sequences (CDS). Hits represented a total of 1018 unique genes, of which 835 (82.02 %) genes were successfully annotated using BLASTX. Subsequently, the KEGG pathway approach for higher-order functional annotation was implemented in DAVID. Using zebrafish as reference genome, a total of 616 zebrafish genes homologous to European eel were mapped to KEGG pathways. Enriched KEGG pathways using a standard setting of gene count = 2 are summarized in Table 6. The pathways with the highest number of genes were MAPK/Erk signalling, calcium signalling, focal adhesion, cell adhesion and GnRH signalling. A list of all annotated genes is provided in Additional file 1: Table S1.

**Table 6:** Over-represented KEGG pathways when comparing glass eels and silver eels from Valencia (Spain), including gene count

Term	Count
dre04010:MAPK/Erk signalling pathway	10
dre04510:Focal adhesion	9
dre04020:Calcium signalling pathway	8
dre04514:Cell adhesion molecules (CAMs)	7
dre04912:GnRH signalling pathway	7
dre04512:ECM-receptor interaction	6
dre04270:Vascular smooth muscle contraction	6
dre04530:Tight junction	6
dre00230:Purine metabolism	6
dre04080:Neuroactive ligand-receptor interaction	6
dre04070:Phosphatidylinositol signalling system	5
dre04914:Progesterone-mediated oocyte maturation	5
dre04910:Insulin signalling pathway	5
dre00564:Glycerophospholipid metabolism	4
dre00562:Inositol phosphate metabolism	4
dre04370:VEGF signalling pathway	4
dre04540:Gap junction	4
dre04210:Apoptosis	4
dre04916:Melanogenesis	4
dre04810:Regulation of actin cytoskeleton	4
dre00534:Heparan sulfate biosynthesis	3
dre00480:Glutathione metabolism	3
dre04650:Natural killer cell mediated cytotoxicity	3
dre04620:Toll-like receptor signalling pathway	3
dre04012:ErbB signalling pathway	3
dre00240:Pyrimidine metabolism	3
dre04350:TGF-beta signalling pathway	3
dre04114:Oocyte meiosis	3
dre04120:Ubiquitin mediated proteolysis	3
dre04144:Endocytosis	3
dre00590:Arachidonic acid metabolism	2
dre00450:Selenoamino acid metabolism	2
dre00250:Alanine, aspartate and glutamate metabolism	2
dre00030:Pentose phosphate pathway	2
dre00270:Cysteine and methionine metabolism	2
dre04621:NOD-like receptor signalling pathway	2
dre00310:Lysine degradation	2
dre00330:Arginine and proline metabolism	2
dre04150:mTOR signalling pathway	2
dre04622:RIG-I-like receptor signalling pathway	2
dre04920:Adipocytokine signalling pathway	2
dre00010:Glycolysis / Gluconeogenesis	2
dre04260:Cardiac muscle contraction	2
dre04520:Adherens junction	2
dre04630:Jak-STAT signalling pathway	2
dre04142:Lysosome	2
dre03040:Spliceosome	2

We also tested for outliers between silver eels and glass eels from Burrishoole, although adult sample size was low (N = 10), as a way to confirm the results above. A total of 2228 putative SNPs under selection were identified by LOSITAN and 1888 SNPs by BAYESCAN, the latter being all included in the list of LOSITAN outliers. Among all outlier SNPs, a hit with a gene was obtained for 949 of the SNPs, 838 corresponding to introns and 111 to exons (including 42 in CDS). In total, 770 (81.13 %) of the hits were successfully annotated using BLASTX. Subsequently, a total of 557 zebrafish genes homologous to European eel were mapped to KEGG pathways in DAVID. The pathway with the highest number of genes was endocytosis (15 genes), while other highly-represented pathways included regulatory and signalling pathways (Table 7). Importantly, despite the limited number of RAD-sequenced adults from Burrishoole, over-represented pathways were similar to the ones found when comparing silver eels and glass eels from Valencia. Shared over-represented pathways included signalling (i.e. MAPK/Erk, GnRH, calcium or insulin) pathways and cell and focal adhesion. A list of all annotated genes is provided in Additional file 2: Table S2.

**Table 7:** Over-represented KEGG pathways when comparing glass eels and silver eels from Burrishole (Ireland), including gene count

Term	Count
dre04144:Endocytosis	15
dre04514:Cell adhesion molecules (CAMs)	8
dre04510:Focal adhesion	8
dre04010:MAPK/Erk signalling pathway	8
dre04270:Vascular smooth muscle contraction	7
dre04912:GnRH signalling pathway	7
dre04020:Calcium signalling pathway	7
dre04080:Neuroactive ligand-receptor interaction	7
dre04060:Cytokine-cytokine receptor interaction	6
dre04142:Lysosome	6
dre04310:Wnt signalling pathway	6
dre04810:Regulation of actin cytoskeleton	6
dre04512:ECM-receptor interaction	5
dre04630:Jak-STAT signalling pathway	5
dre04916:Melanogenesis	5
dre03040:Spliceosome	5
dre04120:Ubiquitin mediated proteolysis	5
dre04530:Tight junction	5
dre00051:Fructose and mannose metabolism	4
dre04540:Gap junction	4
dre04012:ErbB signalling pathway	4
dre04910:Insulin signalling pathway	4
dre04110:Cell cycle	4
dre00512:O-Glycan biosynthesis	3
dre00520:Amino sugar and nucleotide sugar metabolism	3
dre00564:Glycerophospholipid metabolism	3
dre04920:Adipocytokine signalling pathway	3
dre04070:Phosphatidylinositol signalling system	3
dre04520:Adherens junction	3
dre00290:Valine, leucine and isoleucine biosynthesis	2
dre00531:Glycosaminoglycan degradation	2
dre00532:Chondroitin sulfate biosynthesis	2
dre00534:Heparan sulfate biosynthesis	2
dre00640:Propanoate metabolism	2
dre00620:Pyruvate metabolism	2
dre00270:Cysteine and methionine metabolism	2
dre04130:SNARE interactions in vesicular transport	2
dre00970:Aminoacyl-tRNA biosynthesis	2
dre04622:RIG-I-like receptor signalling pathway	2
dre04330:Notch signalling pathway	2
dre04340:Hedgehog signalling pathway	2
dre03320:PPAR signalling pathway	2
dre00562:Inositol phosphate metabolism	2
dre00010:Glycolysis / Gluconeogenesis	2
dre04650:Natural killer cell mediated cytotoxicity	2
dre04620:Toll-like receptor signalling pathway	2
dre04914:Progesterone-mediated oocyte maturation	2

**Fig. 1:**
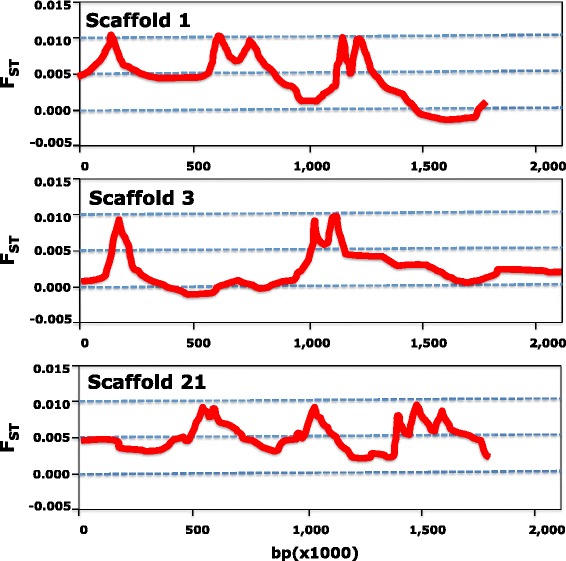
Plots of average F_ST_ calculated using a 50-kb sliding window for the three largest scaffolds (1, 3 and 21) in the European eel genome

Finally, average F_ST_ values between silver eels and glass eels from Valencia calculated using a 50-kb sliding window were plotted for the 30 largest scaffolds. F_ST_ was low throughout the scaffolds, with just a few narrow peaks. No regions of the scaffolds with pronounced divergence peaks were observed, consistent with panmixia removing any effect of diversifying selection from each new generation. Similar results were obtained when using alternative (100-kb and 200-kb) sliding windows. Figure 1 shows an example of the plots obtained for the three largest scaffolds (1, 3 and 21) in the European eel draft genome. A total of 4, 5 and 6 peaks representing outlier SNPs with higher F_ST_ relative to the background were observed in scaffolds 1, 3 and 21, respectively. The average distance between outlier SNPs was around 300,000 bp in scaffolds 1 and 3 and around 220,000 bp in scaffold 21. The closest distance between outlier SNPs was 9000 bp in scaffold 1, 19,000 bp in scaffold 21 but around 125,000 bp in scaffold 3.

## Discussion

### Evidence for selection acting upon different life stages in European eel

We examined the patterns of genomic diversity across life cycle stages in European eel, using a combined approach of candidate coding-gene SNPs and a large-scale genomic analysis of 153,423 SNPs generated by RAD sequencing. We compared two life cycle stages separated by metamorphosis, glass eels (juvenile stage) and silver eels (adult stage), and identified signatures of directional selection. All available information indicates that eel mortality in nature is very high and only a small fraction of the glass eels entering European coasts reach the silver eel stage and migrate back to the Sargasso Sea [25]. Bonhommeau et al. [26] estimated a glass eel survival rate of 10 %, while Åström and Dekker [14] estimated a natural mortality rate of M = 0.14 per year and a fishery mortality rate of F = 0.54 per year. Hence, the observation of outlier loci showing high genetic differentiation when comparing glass eels vs. silver eels is consistent with the action of natural selection acting upon eels. F_ST_-based outlier tests are based on the detection of loci that show significantly high differentiation with significance determined by simulations assuming specific population structure models [27] and are being extensively used at present in studies aiming at detecting signatures of selection on a genome scale [28–31], although results should be interpreted with caution (see Bierne et al. [32] for a critique of outlier tests).

Using the SNP panel developed by Gagnaire et al. [15] in American eel, a total of four loci showed higher genetic differentiation than the background F_ST_ when comparing glass eel and silver eel samples. GAPDH (Glyceraldehyde 3-phosphate dehydrogenase) is a gene with a major metabolic function and catalyzes the conversion of glyceraldehyde 3-phosphate to D-glycerate 1,3-biphosphate in the glycolysis pathway. In this sense, GAPDH has been linked to growth differences in gene expression studies in fishes [33]. CST (Cystatin precursor) is a gene involved in catalytic activity that takes part in defense response. CLIC5 (Chloride intracellular channel 5) is a gene involved in chloride ion transport, which is important for pH regulation, volume homeostasis, organic solute transport, cell migration, cell proliferation and differentiation. CSDE1 (Cold shock domain-containing protein E1) is a RNA-binding protein involved in regulation of transcription. However, all outliers were location-specific (GAPDH in Iceland, CLIC5 in Spain and CST and CSDE in Ireland) and none was common across locations. This suggests that if the outliers detected indeed do represent selection, then they do not result from a universal selection agent affecting eels in all places but rather location-specific factors affecting eels locally.

A much larger number of candidate genes putatively under selection were identified after screening a total of 153,423 RAD-generated SNPs across the genome: 2413 when comparing glass and silver eels from Spain and 2228 in the case of Ireland. Functional annotation analysis using DAVID identified signal transduction pathways as the most over-represented. Signal transduction involves the binding of an extracellular signalling molecule (ligand) to a specific cell-surface receptor. The activation of the receptor leads to an altered response inside the cell. Examples of cellular responses to extracellular stimulation that require signal transduction include changes in metabolism and gene expression. Following are some major pathways identified in our study:

- MAPK/Erk signalling: a complex key signalling pathway that is involved in the regulation of normal cell proliferation, survival, growth and differentiation [34]; the pathway includes mitogen-activated protein kinases that ultimately activate transcription factors and alter gene transcription.

- GnRH signalling: secretion of gonadotropin-releasing hormone from the hypothalamus acts upon its receptor in the anterior pituitary to regulate the production and release of FSH (follicle-stimulating hormone) and LH (luteinizing hormone); together, FSH and LH regulate many aspects of gonadal function in both males and females, including normal growth, sexual development and reproductive function [35].

- Calcium signalling: calcium ions serve a number of important functions and regulate processes as diverse as fertilization, development, learning and memory, mitochondrial function, muscle contraction and secretion; calcium ions are also recognized as very important in ion exchange and osmoregulation [36].

- Insulin-like growth factor signalling: this pathway plays a central role in the neuroendocrine regulation of animal growth and development. In fish, additional functions include osmoregulatory acclimation, reproductive development and tissue regeneration. Insulin-like growth factor signalling is mediated by two ligands, insulin-like growth factor 1 (IGF-1) and factor 2 (IGF-2), both of which were identified as outliers. The production of IGF-1 is stimulated by growth hormone and is positively correlated with growth as shown in coho salmon [37] or sea bream [38].

- Focal adhesion: Related to signalling pathways, focal adhesion was also over-represented in our analysis. Focal adhesions are large protein assembles through which both mechanical force and regulatory signals are transmitted and have central roles in cell migration and morphogenesis as well as regulating cell proliferation and differentiation [39].

Other over-represented pathways were related to metabolism (i.e. purine metabolism, glycerophospholipid metabolism) and detoxification of xenobiotics (i.e. glutathione metabolism). The latter included cytochrome CYP2J6, a member of the cytochrome P450 superfamily of enzymes that catalyze many reactions involved in drug metabolism.

Overall, when we consider the functions of the genes, it seems biologically plausible that the genes identified as outliers are indeed under selection. Many of the over-represented pathways were related to growth, while others could result from the different conditions and habitats that eels inhabit throughout their life cycle. In this sense, examination of otolith data suggests a high plasticity of habitat use by eels [9], with one or several movements between fresh and brackish waters throughout the lifetime of an individual. When only a single habitat switch event was detected, it occurred between 3 and 5 years of age, which could explain the differences found in our study at osmoregulation genes between glass eels and silver eels.

When comparing across methods, a much larger number of genes putatively under selection were identified using the RAD genome scan approach. This is expected, since we screened only 80 SNPs with the American eel SNP panel, while we interrogated over 150,000 SNPs with the RAD approach. However, when considering percentage rather than total number of markers under selection, results were similar. For instance, in the case of Valencia we found 1.57 % of SNPs putatively under selection using the RAD approach (2413 out of 153,423 SNPs screened), with a similar percentage (1.25 %, 1 outlier out of 80) found using the American eel SNP array. In the case of Burrishoole, candidate SNPs under selection were 1.45 % (2228 out of 153,423) using the RAD approach and 2.5 % (2 out of 80) using the American eel SNP array. Finally, it should be noted that outliers were not shared across methods, which is explained by the different nature of the markers. All SNPs included in the array were located in coding-genes, while the SNPs discovered using the RAD approach were mostly located in non-coding genes, since RAD tags are restriction site generated markers randomly distributed across the genome.

### Support for the adaptive decoupling hypothesis

The observation in our study of a large number of outlier loci showing higher F_ST_ values relative to the background when comparing glass eels and silver eels fits the prediction that in the case of animals with complex life cycles, different genes and gene pathways will be under selection at different life stages. This is in accordance with the adaptive decoupling hypothesis for the benefits of metamorphosis [1], which predicts no correlation between traits separated by metamorphosis, thereby each life stage can respond independently to its unique selective pressures.

Moran [1] hypothesized that the genetic decoupling of pre- and post-metamorphosis life stages explains the origin and persistence of complex life cycles, as alternative phases can be regarded as adaptations for a more effective exploitation of resources and adaptations to perform phase-specific tasks. In the case of eels, the silvering metamorphosis, which represents the passage from juvenile to adult, is accompanied by drastic modifications at the physiological, morphological and ecological level that could explain the shifts in selection acting across life cycle stages. Changes occur both externally (increase in eye size, change in colour from yellow-ish to silver, increase in body size and weight) and internally (degeneration of the digestive track, changes of visual pigments, development of gonads). While the juvenile stage is perfectly adapted for feeding, adults are not able to feed anymore following metamorphosis, relying solely on fat reserves to reach the Sargasso Sea. However, the adult stage is best adapted for migration, mating and reproducing.

If selection were complementary between life cycle stages separated by metamorphosis, the same genes and pathways would be expected to be under selection before and after metamorphosis and no outlier loci showing high differentiation would be expected. By contrast, in our study we found up to 2413 outlier loci, suggestive of opposing selection between life cycle stages, as predicted by the adaptive decoupling hypothesis.

Alternatively, results in our study could reflect changes in genetic frequencies over time rather than a selection effect. One possibility we cannot rule out is a temporal effect, since juveniles and adults in our sampling were not from the same cohort. Following the same cohort in time would be ideal to test our hypothesis of opposing selection during different life stages separated by metamorphosis, however this is virtually impossible in eels. Age at maturity (at which the silvering metamorphosis occurs) is highly variable in eels, ranging from 6 to 20 years or more [7]. This means that individuals from the same cohort will become silver eels at different times and that adult eel samples for genetic studies are mostly made up of a mix of different cohorts. Nevertheless, the observation of a parallel pattern of genetic differentiation in Valencia and Burrishoole when comparing glass eels and silver eels, with many over-represented genes and pathways being shared by the two locations, lends support to a selection effect.

### Genomic distribution of candidate genes under selection

Genomic regions displaying elevated differentiation relative to the rest of the genome (genomic islands of divergence) have been described in many species, as exemplified in the case of sticklebacks [28, 29]. In marine fishes, genomic islands of divergence might be unexpected because of their extremely high effective population sizes (N_e_). Due to the effects of N_e_ on the level of linkage disequilibrium, a fast decay of linkage disequilibrium should be generally expected (reviewed in [40]), which in turn might preclude hitchhiking and ultimately the observation of genomic islands of divergence.

That might be the case of eels. In our study comparing glass eels and silver eels, outlier SNPs showing high genetic differentiation consistent with selection did not group into clusters but were generally spread across the genome. Similarly, no apparent genomic islands of divergence were found when investigating the genomic distribution of outlier SNPs between European and American eel [41].

In contrast to the pattern observed in European eel, genomic clustering of some highly divergent SNPs was observed in Atlantic herring [30], a species with a very large effective population size. Elevated genomic differentiation across large genomic blocks (up to 15 Mb) was also reported in Atlantic cod [31, 42], which suggests that genomic islands of divergence can occur in marine fishes. However, it is likely that the different pattern observed in eels (no clustering of genes) vs. Atlantic herring and Atlantic cod (clustering of genes) might be due to the different impact of evolutionary forces acting upon the panmictic European eel and other species that are genetically sub-structured. Therefore, unlike in eels, significant linkage disequilibrium might occur in Atlantic herring and Atlantic cod, thus allowing hitchhiking to accumulate, which ultimately results in the clustering of genes with localized elevated differentiation relative to the background.

Finally, it should be noted that by using an F_ST_-outlier approach, the number of loci under selection might be underestimated. While standard tests of selection (i.e. outlier tests) are powerful tools to detect “hard selective sweeps”, in which a new advantageous mutation arises and spreads quickly to fixation due to natural selection [43], other scenarios might be more difficult to detect. Those include soft sweeps, in which an allele already present in the population (i.e. standing variation) becomes selectively favoured or when multiple independent mutations at a single locus are all favoured [44], and polygenic adaptation, in which simultaneous selection occurs on variants at many loci [22, 45]. Both scenarios lead to shifts in allele frequencies rather than fixation, thus tend to be more difficult to detect than hard sweeps using standard tests of selection [23, 45, 46]. Considering the high historical effective population size estimated for the European eel (from 100,000 to 1×10^6^ individuals) and associated high genetic variability [8], soft sweeps might be more common than hard sweeps and hence our study might have uncovered only a fraction of the genes under selection across life stages in European eel.

## Conclusions

Our data supports the adaptive decoupling hypothesis for the benefits of metamorphosis in European eel since genes and gene pathways under selection were different in pre- and post-silvering metamorphosis. The differences found between juveniles and adults suggests that partitioning the life cycle into discrete stages may be more effective than a single stage in the case of eels. This way,each life stage can perform specific tasks more effectively, i.e. feeding in glass eels, reproducing in silver eels.

## Methods

### Ethical statement

No experiments were conducted on the animals and animal manipulation was limited to sacrificing fish, using the least painful method to obtain tissue samples for DNA extraction. In all cases, in order to minimize the suffering of the animals used in the study, fish were deeply anaesthetized with MS-222 (3-amonobenzoic acid ethyl ester) or 2-phenoxyethanol 1 % and then painlessly sacrificed. All procedures were conducted by technical staff, who had all the necessary fishing and animal ethics permits. In Iceland, sampling was approved by Holar University College Ethical Committee and conducted according to guidelines and laws on animal welfare in Iceland. In Ireland, all sampling was carried out under authorisation (Sec. 4) of the Fisheries Act 1959-2003 by permission of the Department of Agriculture, Food and Marine. In Spain, samples were obtained from professional fishermen with sampling and ethical treatment of animals approved by the Consejeria de Medio Ambiente of the Comunidad Autonoma de Valencia.

### Sampling

A total of 113 silver eels (adult stage) were collected at three locations across the geographical distribution of the species: (i) Valencia (Spain) in the Mediterranean Sea; (ii) Burrishoole (Ireland), in the North Atlantic Ocean; and (iii) four separate sampling sites in southwestern Iceland that were pooled to increase sample size (Table 8). All silver eels were caught using fyke nets. Silver eels were compared to previously analyzed glass eels collected in the same locations [11, 16]. We also used previously analyzed American eels for comparison [8, 16]. Genomic DNA was extracted using standard phenol-chloroform extraction.

**Table 8:** Sampling details including sampling date and locations and number of individuals genotyped using the American eel SNP chip and number of individuals RAD sequended in all glass eel and silver eel samples

Country	Location	Coordinates	Sampling date	N-chip	N- RAD
1) Glass eels					
Spain	Valencia	39° 46’ N / 0° 24’ W	2010	44	31
Ireland	Burrishoole	53° 90’ N / 9° 58’ W	2005	39	29
Iceland	Stokkseyri	63° 81’ N / 21° 04’ W	2001	10	-
	Vifilsstadvatn	64° 07’ N / 21° 87’ W	2001	10	-
	Seljar	64° 56’ N / 22° 31’ W	2001	10	-
	Vogslækur	64° 69’ N / 22° 33’ W	2001	10	-
2) Silver eels					
Spain	Valencia	39° 46’ N / 0° 24’ W	2010	40	31
Ireland	Burrishoole	53° 90’ N / 9° 58’ W	2010	40	10
Iceland	Vatnsdalsá	65° 49’ N / 20° 34’ W	2000	9	-
	Grafarvogur	64° 15’ N / 21° 81’ W	2003	10	-
	Vifilsstadvatn	64° 07’ N / 21° 87’ W	2002	9	-
	Grindavik	63° 83’ N / 22° 42’ W	2003	5	-

### SNP genotyping

A panel of 100 coding-gene single nucleotide polymorphisms (SNPs) developed by Gagnaire et al. [15] in American eel was applied to all 113 silver eels in our study (40 from Spain, 40 from Ireland and 33 from Iceland). In a preliminary analysis, 20 out of the 100 primer sets did not give good amplification products in European eel and were excluded. Subsequently, all individuals were genotyped at 80 SNPs [16], using the Kbioscience Competitive Allele-Specific PCR genotyping system (KASPar) (Kbioscience, Hoddeston, UK).

Within-sample genetic diversity was assessed by observed and expected heterozygosities, polymorphism and mean and total number of alleles using GENEPOP [47] and standardized allelic richness using FSTAT [48]. Differences in genetic diversity among samples were tested by one-way ANOVA using STATISTICA (StatSoft Inc). Deviations from Hardy-Weinberg equilibrium and differences in allele frequencies among samples were calculated using GENEPOP. Significance levels for multiple comparisons were corrected using Bonferroni [49].

Prior to the test for selection, we tested the presence of hybrid individuals in the dataset using STRUCTURE [50]. We included a set of 20 American eels for reference and conducted the analysis assuming a K = 2 scenario given that two panmictic species were analyzed. We assumed an admixture model, uncorrelated allele frequencies and we did not use population priors. A burn-in length of 100,000 steps followed by one million additional iterations was performed.

### RAD sequencing

A subset of 41 silver eels (31 from Spain and 10 from Ireland) were RAD-sequenced [51, 52] at BGI (Beijing Genomics Institute, Hong Kong). In short, genomic DNA for each individual was digested with restriction enzyme EcoRI, ligated to a modified Illumina P1 adapter containing individual-specific barcodes and sheared to an average size of 500 bp. Sheared DNA was separated by electrophoresis on a 2 % agarose gel and fragments in the 350-500 bp size range were selected. After treating dsDNA ends with end blunting enzymes and adding 3’-adenine overhangs, a modified Illumina P2 adapter was ligated. The final step consisted in enriching the libraries by PCR amplification. The libraries for the 41 silver eels were constructed together with those for 259 glass eels reported in Pujolar et al. [11], using the same methodology and conditions. RADs for each individual were sequenced (10 individuals per sequencing lane) on an Illumina Genome Analizer II.

The analysis of the RAD data was conducted simultaneously for the 41 silver eels plus 60 glass eels (31 from Valencia and 29 from Burrishoole) that were first analyzed in Pujolar et al. [11] but were re-analyzed together with the silver eels. This way, the same software and parameters were used for filtering, alignment to the eel genome and SNP discovery, i.e. using the same version of Stacks (version 1.09). Gene annotation and functional annotation analysis were also conducted simultaneously for silver eels and glass eels.

The 90 bp-long RAD sequences obtained from the Illumina runs were sorted according to barcode and quality filtered using the FASTX-Toolkit [53]. Criterion for quality filtering was that all nucleotides positions must have a minimum Phred score of 10, otherwise the read was discarded. Final read length was trimmed to 75 nucleotides in order to minimize sequencing errors usually found at the tails of the sequences [8].

Quality-filtered reads were then aligned to the European eel draft genome (www.eelgenome.com) using the un-gapped aligner BOWTIE [54]. A maximum of two mismatches between reads and genome were allowed. In order to avoid paralogs, reads with alternative (two or more) alignments to the genome were excluded.

Assembly of RAD sequences into loci and SNP identification were performed using the ref.map.pl pipeline in Stacks version 1.09 [55]. First, pstacks was used to align exactly-matching sequences into stacks that were subsequently merged to form putative loci. At each locus, nucleotide positions were examined and SNPs were called using a maximum likelihood framework. A minimum stack depth of 10 was used. Second, cstacks was used to build a catalog of all existing loci and alleles after merging loci from multiple individuals. Third, sstacks was used to match all individuals against the catalog. Finally, the program Populations in Stacks was used to process all SNP data across individuals. The minimum percentage of individuals in a population required to process a locus was set to 66.67 %.

Prior to the SNP analysis, loci in the catalog were further filtered in order to remove paralogs and otherwise spurious loci according to the following three criteria: exclude loci with extremely higher coverage as it might indicate the presence of more than one locus (threshold used was twice the standard deviation from the mean number of reads); exclude tri-allelic loci since the presence of more than two alleles might result from sequencing errors; exclude loci containing SNPs with observed heterozygosity (H_o_) of 1 (all individuals genotyped were heterozygotes) or 0 (all individuals homozygotes), suggestive of the presence of more than one locus.

Measures of genome-wide genetic diversity, including observed and expected heterozygosities and nucleotide diversity were calculated in Stacks. Differences in genetic diversity among samples were tested by one-way ANOVA using STATISTICA. Deviations from Hardy-Weinberg equilibrium and genetic differentiation were calculated in GENEPOP.

We also tested for hybrids using a subset of species-specific diagnostic SNPs (F_ST_ = 1) between European and American eel [56]. The analysis in STRUCTURE included RAD sequenced individuals in this study together with a sample of 30 RAD-sequenced American eels for comparison. STRUCTURE was run following the parameters described above.

### Identification of candidate SNPs under selection

Candidate SNPs for being under directional selection were identified using two different outlier tests. First, we used the selection detection workbench LOSITAN [57], which uses a coalescent-based simulation approach to identify outliers based on the distributions of heterozygosity and F_ST_ [58]. A neutral mean F_ST_ was enforced by removing potentially non-neutral loci after calculating an initial mean F_ST_, as recommended by Antao et al. [57]. We used a very strict threshold of 0.995 and a 10 % false discovery rate to minimize thte number of false positives. Second, outlier SNPs were also detected using BAYESCAN [59], a Bayesian method based on a logistic regression model that separates locus-specific effects of selection from population-specific effects of demography. BAYESCAN runs were implemented using default values for all parameters, including a total of 100,000 iterations after an initial burn-in of 50,000 steps. Posterior probabilities, q values and alpha coefficients were calculated. A q-value of 10 % was used for significance.

### Candidate gene annotation

Genomic position of the candidate SNPs for local selection were established on the basis of the gene predictions for the European eel genome (http://www.zfgenomics.org/sub/eel) using a custom-made script [11]. SNPs were considered to be located in a gene when included in CDS (complete coding sequences), exonic and intronic regions. Functional annotation of those genes was obtained using Blast2Go [60], which conducts BLAST similarity searches and maps GO (Gene Ontology) terms to the homologous sequences found. Only ontologies with E-value < 1E-6, annotation cut-off > 55 and a GO Weight > 5 were considered for annotation. Additionally, functional interpretation of the set of candidate genes was obtained using the DAVID (Database for Annotation, Visualization and Integrated Discovery) web-server v6.7 [61]. We conducted the analysis in DAVID to establish whether several genes were associated with the same function or pathway and therefore facilitate interpretation of our results, regardless of statistical significance. The zebrafish (*Danio rerio*) genome was used as reference for annotation. Prior to the analysis in DAVID, a local BLAST was conducted for significant matches directly against zebrafish Ensembl proteins using BLASTX. Zebrafish Ensembl Gene IDs were obtained from the corresponding Ensembl protein entries using the Biomart data mining tool [62]. Gene functional analysis in DAVID was conducted defining the zebrafish IDs corresponding to those genes including a locally selected SNP as ‘Gene list’ and the zebrafish IDs corresponding to all genes as ‘Background’. Standard settings of gene count = 2 and ease = 0.1 were used.

Finally, patterns of differentiation across genome regions were characterized to test whether genes putatively under selection were grouped into clusters (genomic islands of differentiation) or more scattered across the genome. We estimated levels of genetic differentiation between glass eels and silver eels in Valencia by calculating average F_ST_ for 50-kb genomic sliding windows. Alternative sliding windows (100 and 200-kb) were also tested. Windows were restricted to the 30 largest scaffolds (903,936 – 2,025,234 bp) from the European eel draft genome.

### Availability of supporting data

The data set supporting the results of this article is available from Dryad: http://datadryad.org/resource/doi:10.5061/dryad.kc1q1. All sequencing files (.fastq) can be found on the NCBI´s Sequence Read Archive under accession number SAMN03786011.

## Additional files

Additional file 1: Table S1. List all annotated genes detected as outliers when comparing silver eels and glass eels from Valencia (Spain) using the RAD approach. Additional file 2: Table S2. List all annotated genes detected as outliers when comparing silver eels and glass eels from Burrishoole (Ireland) using the RAD approach.
